# Effects of Functional Fitness Enhancement through Taekwondo Training on Physical Characteristics and Risk Factors of Dementia in Elderly Women with Depression †

**DOI:** 10.3390/ijerph18157961

**Published:** 2021-07-28

**Authors:** Sun-Hong Baek, Ga-Ram Hong, Do-Kyum Min, Eun-Hee Kim, Sang-Kab Park

**Affiliations:** 1 Taekwondo Institute of Health and Culture, Dong-A University, 37, Nakdong-Daero, 550, Sahagu, Busan 49315, Korea; 87sunhong@naver.com (S.-H.B.); hgr2002@naver.com (G.-R.H.); 2 Graduate School, Dong-A University, 37, Nakdong-Daero 550, Sahagu, Busan 49315, Korea; dkmin9180@donga.ac.kr; 3 Department of Taekwondo, Dong-A University, 37, Nakdong-Daero 550, Sahagu, Busan 49315, Korea; 4 College of Arts and Sports, Dong-A University, 37, Nakdong-Daero, 550, Sahagu, Busan 49315, Korea

**Keywords:** Taekwondo training program, depression, elderly women, functional fitness, physical characteristics, dementia risk factor, serum lipids

## Abstract

The purpose of this research is to identify the correlations between functional fitness enhancement through a long-term Taekwondo training program and the physical characteristics and risk factors of dementia among elderly women with depression. The study has found that conducting three 60-min Taekwondo training sessions a week for the duration of 12 weeks has enhanced a number of functional fitness indexes, including hand grip strength/weight (*p* < 0.01), 4-m gait speed (*p* < 0.001), 3-m timed up and go (*p* < 0.05), and figure-of-eight track (*p* < 0.05), and significantly improved general health condition indexes as well, including percent fat (*p* < 0.05), appendicular skeletal muscle mass index (*p* < 0.01), systolic blood pressure (*p* < 0.01), and diastolic blood pressure (*p* < 0.001). Furthermore, the arteriosclerosis index and cognitive function have been found to be improved with an increase of brain-derived neurotrophic factor (BDNF; which prevents dementia) and a significant decrease of β-amyloid—a risk factor of dementia—as a result of enhancements in serum lipids and adiponectin, confirming the positive effects of functional fitness enhancement on fighting depression, promoting physical characteristics, and reducing the risk factors of dementia.

## 1. Introduction

The rapidly aging society has contributed to a sharp increase in the number of dementia patients. According to the 2017 Survey of Dementia Prevalence conducted by Statistics Korea, one in ten elderly have dementia. In particular, one in three individuals aged 85 years or over are reported to have dementia [1,2]. Moreover, the physical, financial, and emotional burdens experienced by the caregiving family members for dementia patients all contribute to making this a serious social issue [3].

While the precise underlying mechanism of dementia remains unclear [4], the causal factors of dementia have been reported to include lifestyle, stress, low physical activity, and chronic illness [5,6].

Especially, increasing β-amyloid has been reported to degrade the cognitive function and to cause neurodegenerative disorders [7]. In addition, irisin, a myokine secreted from muscle as it contracts, has been known to convert white fat into brown fat, allowing the adipose tissues to generate more heat and to consume more energy [8]. Meanwhile, a recent study has reported its critical role in energy metabolism as well as adaptating during exercise and in promoting the release of brain-derived neurotrophic factor (BDNF) [9]. With a neurotrophic factor originating from the brain, BDNF is essential in maintaining brain health as a substance that induces nerve cell growth [10].

Meanwhile, the functional fitness of older people is defined as the physical ability to perform daily routines in a safe and independent manner [11]. In particular, the hand grip strength, as one of the functional fitness parameters, is used to assess the aging process and chronic illnesses among older people [12], and is reported to show a high correlation with diabetes, metabolic syndrome, and mortality as well [13,14,15,16,17,18,19].

Depression among the elderly has also been reported to be closely correlated with dementia [20,21,22], which is considered highly important with regard to the changes in the functional fitness of the elderly. Thus, a number of lifestyle interventions have been suggested [23], one of which is regular exercises that help maintain the cognitive function and reduce the potential of dementia among the elderly [24,25].

As one of the previous studies related to exercises, Lee [26] investigated the relationship between the symptoms of depression and physical functions in older women and reported that the depression score (GDS–K) decreases as physical functions, such as hand grip strength and lower limb muscle strength increase. Damirchi et al. [27] found that the people aged 65 years and over with mild cognitive impairment enhanced their cognitive functions in terms of the BDNF expression, synaptic plasticity, learning memory, and neurological development after they were given an 8-week mental training program.

Park [28] conducted a study where older women with metabolic syndrome were encouraged to participate in a 12-week Taekwondo program comprised of three sessions per week and 60 min per session, and found its positive effect on the health-related fitness and an improvement in insulin, Homeostasis Model Assessment of Insulin Resistance(HOMA-IR), and metabolic syndrome. In addition, Lee et al. [29] examined older women with high blood pressure who participated in the 12-week Taekwondo program comprised of 60 min per session and three sessions per week, and found an improvement in the blood pressure and cardiovascular functions following the program.

As such, Taekwondo offers the unique advantage for individuals to adjust the exercise intensity and movements based on his/her physical characteristics but has seen little research on its functional fitness and dementia risk factors among elderly women with depression.

In this respect, this study seeks to identify the effect of functional fitness enhanced by Taekwondo training on the physical characteristics and dementia risk factors among elderly women with depression.

## 2. Materials and Methods

### 2.1. Study Subjects

To determine the sample size, the G*Power version 3.1.9.2 statistical power analysis software program has been used. The estimated sample size required to obtain a minimum power of 80% at a significant alpha of 95% is 12. According to a prior pilot test, at least 12 participants are required in each of the two study groups. The alpha level is 0.05.

Twenty-four senior citizens aged 65 or older have been recruited to participate in the study by posting ads on the bulletin boards of various welfare centers managed by the Sarangchae Senior Welfare Center in Busan, South Korea. Participants have signed a prior consent to participate in the study and have been assigned randomly to either one of the two groups by using a computer program with unique numbers given according to the alphabetic order of names on the list (https://www.randomizer.org (site accessed on 20 March 2019): Taekwondo Group (*n* = 12) and Control Group (*n* = 12). Four of them were dropped from the study for personal reasons. In addition, the measurements have been made on the Taekwondo training group followed by the control group with notifications sent to the participants in both Taekwondo training group and the control group upon completion of the preliminary measurement.

The eligibility criteria include the appendicular skeletal muscle mass index (ASMI) ≤ 5.7 kg/m^2^ and geriatric depression scale-Korea (GDS–K) ≥ 14-point. In addition, the participants have been instructed to maintain their diet as usual during the training for 12 weeks. The participants’ physical characteristics are presented in Table 1.

The study has been conducted with the approval of the institutional review board (IRB) at the Dong-A University (2-104 0709-AB-N-01-201904-HR-023-02).

### 2.2. Methods

#### 2.2.1. Physical Examination

An aneroid sphygmomanometer (KENCO, CK-E301, Taipei, Taiwan) and a body composition analyzer (Inbody 470, Biospace Co., Ltd., Seoul, Korea) have been used to measure the blood pressure and body height, body weight, body fat percentage, and lean body mass, respectively. The body mass index (BMI) is calculated as a person’s weight in kilograms divided by the square of height in meters was calculated using the body weight and body height equation (kg/m^2^). In addition, the appendicular skeletal muscle mass (ASM) was measured, and the ASMI, ASM/m^2^, was calculated by bioimpedance analysis (BIA).

#### 2.2.2. Functional Fitness

To measure the functional fitness, the necessary revision and complementation have been made based on the senior fitness parameter of the Korea Sports Promotion Foundation [30]. The speed and reaction time have been measured by the 4 m gait speed; power through hand grip strength and sit-to-stand for 30 s; agility and coordination through a figure-of-eight walk test; flexibility through a sitting forward bend; and a3m timed up and go has been used to test the equilibrium.

#### 2.2.3. Geriatric Depression Scale-Korea

To examine the depression levels of the elderly, the GDS-K, which is the standardized version by Jung et al. [31] of the original GDS developed by Yesavage et al. [32] has been used. The GDS-K consists of a total of 30 questions: 16 negative and 14 positive ones. The maximum score is 30, with a score ≥ 22 indicating severe depression, 19–21 moderate depression, 14–18 mild depression or suspected depression, and a score ≤ 14 indicating a healthy state.

#### 2.2.4. Korean Dementia Screening Questionnaire

The Korean dementia screening questionnaire (K-DSQ) developed by Yang et al. [33] has been used. The questionnaire contains a total of 15 questions in three different categories, including memory impairment, other cognitive functional disorders, and difficulty performing daily activities with the same number of questions for each category. The maximum score is 30, with a score of ≥6 indicating a high probability of dementia (sensitivity 79% and specificity 80%).

#### 2.2.5. Taekwondo Training Program

This study has designed and implemented the Taekwondo training program for 12 weeks with 60 min per session and three sessions per week with an aim to enhance the functional fitness of elderly women, including agility, equilibrium, coordination, power, speed, and reaction time. Agility is the ability to change the position and direction of the body in a rapid and efficient way. For this, the participants have been guided to perform the Seogi moves (Several postures where one stands on both feet to control the distance from the opponent or to perform techniques) of Taekwondo, including Juchumseogi (the posture of both legs standing as if they were a little sitting), Apseogi (the posture of taking a step forward and putting the center of gravity in the middle), Apgubi (the posture of taking a step forward with one’s center of gravity in front), and Dwitgubi (the posture of taking a step back and putting the center of gravity in the back). Upon instruction, the participants performed the motion to change direction (front, back, left, right) while standing with their feet apart in a straight or slightly diagonal line. The participants were also instructed to perform the Makgi moves, (technique of blocking an opponent’s attack with arms), including Naeryeomakgi (technique of any downward cover using the wrist to block the opponent’s attack on the lower part of the body such as abdomen or balls), Momtongmakgi (act of middle blocking nad deflecting away from the attacker by starting up the opposite shoulder and projecting the wrist down and across the body), and Ollyeomakgi (any cover of getting the wrist from down to up against the attack on the face), in which they rapidly rotate their wrists but control them precisely at a target position, to maintain body control and complete the move within a short time. Equilibrium was based on the Balchagi moves (technique of striking the target by kicking or spinning kicks), including Apchagi (technique of raising the knee to the waist, pulling the toes back and quickly extending the foot at the target right in front), Dollyeochagi (technique of turning the leg as well as waist and kicking the target from the outside toward the inside using the top of the foot), and Naeryeochagi (technique of kicking the face or chest of the opponent downward from above using the sole of the foot or bottom of the heel), in which the participants kicked with one leg while maintaining their balance with the other in a repeated manner to enhance the effects of dynamic balance.

Coordination is the ability to integrate two or more movements for accurate, smooth, and harmonious balance of body motion. For this, the participants performed the chapter one of Taegeuk 1 Jang based on the primary moves (Seogi, Makgi, Balchagi, Jireugi) of Taekwondo and the Taekwon Aerobics to the music in the order of the set movements, whereby the participants learned the movements in various forms.

For power and speed, the participants performed the Balchagi moves, including Apchagi, Dollyeochagi, and Naeryeochagi, following the instructor’s command, in a direction to—hit a target position to stimulate the quadriceps and the hip muscle.

In particular Reaction time and speed measure the ability to rapidly thrust the body forward, which contributes to the development of the nervous system. Thus, to promote nervous system development and to improve the speed and reaction time, the participants were instructed to perform the Jireugi moves(techniques that strike or punch the target to give an impact on it whether it is a person or an object) including Momtongjireugi(a fist strike aimed at the mid-section of the opponent by extending the arm straight using the turning force of the body), Ulgooljireugi(a fist strike aimed at the face of the opponent by extending the arm straight using the turning force of the body), Naeryeojireugi(a fist strike aimed at the lower chest of the opponent by extending the arm straight using the turning force of the body), and Yeopjireugi(striking technique that punches from the side using the turning force of the body), while reacting as rapidly as possible to the instructor’s whistle and to control their movements thereafter.

Warm-up and cool-down exercises were comprised of stretches, memory recall, and connecting words in pairs.

The Taekwondo training program is presented in Table 2 [34] with the rating of perceived exertion (RPE) from Borg [35] used for the program intensity.

### 2.3. Blood Sample Analysis

After a 12-h fasting period, a 15 mL blood sample has been collected from the brachial-antecubital vein, left at room temperature at 4 °C for one hour each, and centrifugated at 3000 rpm for 15 min. The sample has been stored at –80 °C for subsequent analyses with the sandwich enzyme-linked immunosorbent assay (EUSA) used for serological analysis.

To measure the total cholesterol (TC), triglyceride (TG), low density lipoprotein cholesterol (LDL-C), high density lipoprotein cholesterol (HDL-C), and free fatty acid (FFA), the enzymatic method has been used. Meanwhile, the arteriosclerosis index has been calculated using the following equation: (TC-HDL-C)/HDL-C). The enzyme-linked immunosorbent assay (ELISA) has been used to analyze the irisin, β-amyloid, and BDNF, and radioimmunoassay (RIA) to analyze the adiponectin. Murine adiponectin with 125I attached to the specimens and multispecies adiponectin rabbit antiserum have been mixed for binding, to which the PEG precipitation solution was added so that the amount of adiponectin could be measured through the bound forms based on the principle of the double antibody method. As reagents, the Human Adiponectin 125I Tubes RIA KIT (Linco Research Inc., Saint Louis, MO, USA) has been used.

### 2.4. Statistical Analysis

The SPSS 24.0 Windows software (IBM, SPSS Statistics 21) has been used to describe all the measured values of all factors as the mean (M) and standard deviation (SD). Prior to the experiment, the homogeneity between the Taekwondo group and the control group has been tested using the Mann-Whitney u *t*-test. A two-way repeated ANOVA has been used to examine interactions between period and group and differences between groups. In addition, to determine the correlation between each factor, the Pearson’s correlation coefficient (r) has been used with the statistical significance set to *p* < 0.05.

## 3. Results

### 3.1. Functional Fitness

The changes in functional fitness between the time before the Taekwondo training program and 12 weeks after are presented in Table 3. 

Between-group and between-time interactions were found for Hand grip strength (*F* = 11.426, *p* < 0.01), Hand grip strength/Weight (*F* = 13.114, *p* < 0.01), 4 m gait speed (*F* = 108.251, *p* < 0.001), 3 m TUG (9.483, *p* < 0.05), and Figure-of-eight track (*F* = 10.027, *p* < 0.05).

### 3.2. Physical Characteristics

The changes in physical characteristics between the time before the Taekwondo training program and 12 weeks after the program are presented in Table 4.

Between-group and between-time interactions were found for body fat percentage (*F* = 9.733, *p* < 0.05), ASMI (*F* = 16.647, *p* < 0.01), systolic blood pressure (*F* = 11.864, *p* < 0.01), diastolic blood pressure (*F* = 24.234, *p* < 0.001), GDS-K (*F* = 29.110, *p* < 0.001), and positive items (*F*= 8.027, *p* < 0.05).

### 3.3. Dementia Risk Factors

The changes in dementia risk factors between the time before the Taekwondo training program and 12 weeks after the program are presented in Table 5.

Between-group and between-time interactions were found for TC (*F* = 31.936, *p* < 0.001), TG (*F* = 23.761, *p* < 0.01), LDL-C (*F* = 25.636, *p* < 0.01), HDL-C (*F* = 21.890, *p* < 0.01), FFA (*F* = 9.871, *p* < 0.05), adiponectin (*F* = 17.272, *p* < 0.01), arteriosclerosis index (*F* = 27.408, *p* < 0.01), K-DSQ (*F* = 19.377, *p* < 0.01), β-amyloid (*F* = 19.314, *p* < 0.01), and BDNF (*F* = 9.854, *p* < 0.05).

### 3.4. Correlations among Hand Grip Strength/Weight, Depression and Dementia Risk Factors

**Figure 1:**
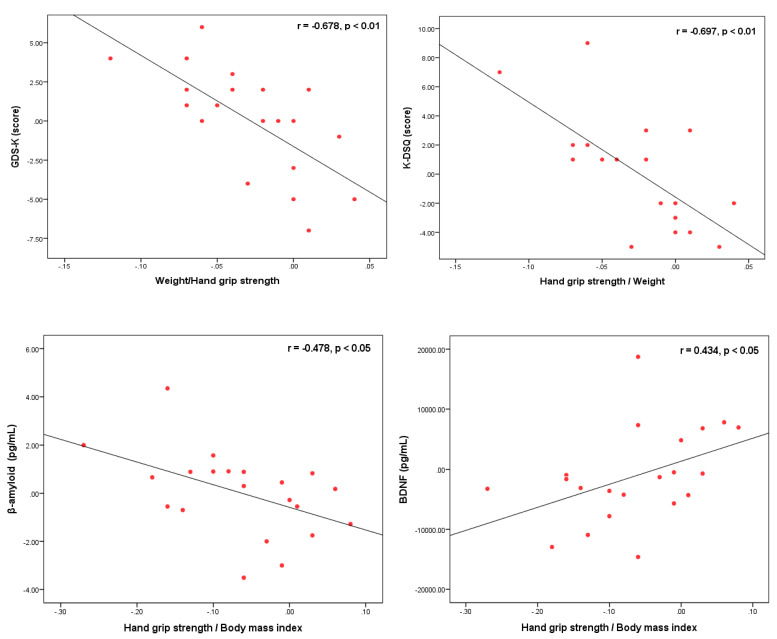
Correlation coefficient between H/W and GDS-K, K-DSQ, β-amyloid, BDNF (*n* = 20). H/W: hand grip strength/Weight, GDS-K: geriatric depression scale Korea, K-DSQ: Korean dementia screening questionnaire, BDNF: brain-derived neurotrophic factor.

The Correlations among hand grip strength/weight, depression and dementia risk factors are illustrated in Figure 1.

The H/W ratio, which is an element of functional fitness is found to have a negative correlation with GDS-K (r = −0.678, *p* = 0.001), K-DSQ (r = 0.697, *p* < 0.01), β-amyloid (r = 0.478, *p* < 0.05) and to have a static correlation with BDNF (r = 434, *p* < 0.05).

## 4. Discussion

Taekwondo training for 12 weeks with three sessions a week and 60 min per session has demonstrated to bring changes to the physical characteristics [Percent fat (*p* < 0.05), ASMI (*p* < 0.01), Systolic blood pressure (*p* < 0.01), Diastolic blood pressure (*p* < 0.01), and GDS-K (*p* < 0.01)] and to enhance the functional fitness as well. Among which, the hand grip strength/weight and relative hand grip strength/weight are reported to have a significant impact on the independent daily life of the elderly [12]. The decrease in these grip strengths is reported to affect the cognitive impairment and dementia [36,37], and 12 weeks of taekwondo training has improved the grip strengths by 15.38 (%diff). Kim et al. [38] reported that Taekwondo intervention has made a significant improvement on the grip strengths of elderly women, further supporting the study findings. This is attributed to the fact that the rather extended period of fist clenching while performing various Taekwondo moves such as Jireugi and Makgi as part of the Taekwondo training are considered to increase grip strengths by improving the forearm muscles. Taekwondo also consists of a variety of basic moves, including Jjumeok Jireugi, Makgi, Balchagi, and Seogi using hands and feet that are easy for the elderly to follow [39] and it is presumed to be an effective exercise program for the elderly in terms of enhancing physical strength, body composition, and muscular strength [40].

Meanwhile Kim et al. [41] reported that lower BMIs among the elderly cause a decline in cognitive function while [42,43,44] reports that the lower the ASMI is, the worse the cognitive function is, making it difficult to maintain independent daily routines such as walking. This study has found that the body fat ratio (*p* < 0.01) has a significant effect and the appendicular skeletal muscle mass index (ASMI) has a statistically significant interaction (*p* < 0.01) while the body mass index does not bring about a significant change. This indicates that Taekwondo training increases the ASMI as various Taekwondo moves involving successive Balchagi and Seogi while standing on one foot, such Ap-chagi, Dollyeo-chagi, and Naeryeo-chagi boosts muscle strength and promote bodily balance, and Baek [25] has reported that higher ASMI is effective in relieving depression symptoms.

In addition, Bartels et al. [45] mentioned that higher symptoms of depression are likely to cause higher prevalence of dementia and that the BDNF enhanced by exercise helps relieves depression [46]. This study has identified the significant interaction effects of BDNF as well as GDS-K (*p* < 0.001), which indicates the functional fitness improved by Taekwondo training is a key cause.

In particular, an increase in BDNF, which may improve dementia risk factors through enhanced functional fitness, is reported to reduce B-amyloid frequently found in the brain of Alzheimer’s patients [47]. Jørgensen et al. [48] reported no significant improvement in BDNF although it has shown an enhancement in muscle strength as a result of resistance exercise while Marinus et al. [49] reported that combining muscle exercise and aerobic exercise is effective in increasing the effects of BDNF. Taekwondo training is believed to enhance muscle strengths as well as cognitive function thanks to the successive moves of both hands and feet at the same time by remembering the basic movements and aerobic/anaerobic poomsae [40].

In addition, irisin, a skeletal muscle-secreted myokine, is known to be expressed and produced by the contraction of muscle, enter the central nervous system through the blood-brain-barrier and to inhibit the cohesion of B-amyloid while promoting BDNF [50,51]. Precedent studies on irisin have focused mainly on high intensity intervention applicable to women in obesity (BMI 30.34 ± 1.27) with the average age of 30.15 ± 2.96 or 90% of the target heart rate (THR) [9,52]. However, this study has identified that the Taekwondo training with moderate intensity of RPE 10-13 among aged women with depression brings out the interaction effects of BDNF (*p* < 0.01) and β-amyloid(*p* < 0.05) despite no significant changes in irisin, which is a meaningful result that has comparatively analyzed the dependent variables of dementia.

This study comes with limitations of having a small population of elderly women only as a single center. Whether changes in the variables observed in this study are a result of Taekwondo training cannot be confirmed. Therefore, following studies should add more people and the elderly to the group to confirm the superiority of Taekwondo and comparative analysis and to verify the effects of Taekwondo training.

## 5. Conclusions

The Taekwondo training program enhanced the functional fitness, leading to an improvement in the depression and physical characteristics of the elderly participants. The correlation with dementia risk factors was thus identified based on the improved cognitive functions and the reduced level of β-amyloid.

## Figures and Tables

**Table 1:** The characteristics of the study subjects.

Variable	Taekwondo (*n* = 10)	Control (*n* = 10)	*p*-Value
Age (years)	72.55 ± 5.45	72.40 ± 3.81	0.918
Body height (m)	1.50 ± 0.03	1.50 ± 0.05	0.645
Body weight (kg)	55.95 ± 4,94	54.12 ± 3.26	0.756
Body mass index (kg/m^2^)	24.72 ± 2.10	24.20 ± 1.98	0.809
Percent fat (%)	42.41 ± 3.78	42.17 ± 5.26	0.973
Systolic blood pressure (mmHg)	148.82 ± 9.93	148.20 ± 14.03	0.863
Diastolic blood pressure (mmHg)	86.91 ± 8.22	83.30 ± 8.72	0.349
ASMI (kg/m^2^)	5.34 ± 0.30	5.55 ± 0.14	0.051
GDS-K (score)	15.64 ± 1.75	16.00 ± 1.76	0.809
K-DSQ (score)	5.73 ± 2.87	5.90 ± 2.88	0.918

ASMI: appendicular skeletal muscle mass index; GDS-K: geriatric depression scale-Korea; K-DSQ: Korean dementia screening questionnaire. Values are means ± standard deviations. *p*-value was analyzed by Mann–Whitney U *t*-test.

**Table 2:** Taekwondo training program.

Item	Contents	Time (min)
Warm-up exercises	Walking at the gym with pairs Memory recollecting and word chaining with pairs Stretching (sit down, spread legs lightly and bend down)	10
Taekwondo training program	1∼6 week’s RPE ^1^; 10∼11	40
	Seogi—Juchumseogi, Apseogi, Apgubi, Dwitgubi, Moaseogi, Naranhiseogi, Hakdariseogi (10 min) Jireugi—Momtongjireugi, Ulgooljireugi, Naeryeojireugi, Yeopjireugi, Jeocheojireugi, Chetdarijireugi, Pyojeokjireugi (10 min) Makgi—Naeryeomakgi, Momtongmakgi, Ollyeomakgi, Momtonganmakgi, Momtongbakkanmakgi, Geumgangmakgi, Santeulmakgi (10 min) Chagi—Ap-chagi (Front, Dollyeo-chagi, Naeryeo-chag. Yeopchagi, Dwichagi, Dwidollyeochagi (10 min)	
	7∼12 week’s RPE; 12∼13	
	Seogi—Juchumseogi, Apseogi, Apgubi, Dwitgubi, Moaseogi, Naranhiseogi, Hakdariseogi (5 min) Jireugi—Momtongjireugi, Ulgooljireugi, Naeryeojireugi, Yeopjireugi, Jeocheojireugi, Chetdarijireugi, Pyojeokjireugi (5 min) Makgi—Naeryeomakgi, Momtongmakgi, Ollyeomakgi, Momtonganmakgi, Momtongbakkanmakgi, Geumgangmakgi, Santeulmakgi (5 min) Chagi—Ap-chagi (Front, Dollyeo-chagi, Naeryeo-chag. Yeopchagi, Dwichagi, Dwidollyeochagi (5 min) Taeguek 1 Jang (separated once, once without a verbal order) (10 min) Taekwon Aerobics (separation action once, once in tune with the music) (10 min)	
Cool-down exercises	Walking at the gym with pairs Memory recollecting and word chaining with pairs Stretching (rolling shoulder and neck to lie down)	10

^1^ Borg [35]; RPE: rating of perceived exertion.

**Table 3:** The changes of functional fitness between the groups at baseline and after 12 weeks.

Variable	Group	Baseline	12 Weeks	Source	*p*-Value
Hand grip strength (kg)	Taekwondo	16.55 ± 1.40	19.09 ± 1.43	Group	0.227
				Time	0.002 **
	Control	17.07 ± 1.49	16.97 ± 1.39	Group × Time	0.008 **
Hand grip strength/weight	Taekwondo	0.30 ± 0.04	0.35 ± 0.04	Group	0.729
				Time	0.000 ***
	Control	0.32 ± 0.03	0.32 ± 0.03	Group × Time	0.006 **
4-m gait speed (m/s)	Taekwondo	0.75 ± 0.05	0.85 ± 0.06	Group	0.011 *
				Time	0.004 **
	Control	0.76 ± 0.03	0.73 ± 0.03	Group × Time	0.000 ***
3-m timed up and go (sec)	Taekwondo	7.00 ± 0.47	6.46 ± 0.70	Group	0.250
				Time	0.274
	Control	7.03 ± 1.09	7.23 ± 1.17	Group × Time	0.013 *
Figure-of-eight track (sec)	Taekwondo	31.08 ± 3.58	26.53 ± 1.95	Group	0.080
				Time	0.003 **
	Control	31.96 ± 4.22	32.01 ± 4.01	Group × Time	0.011 *
30 s chair stand (frequency)	Taekwondo	19.00 ± 3.85	20.64 ± 3.17	Group	0.871
				Time	0.137
	Control	20.10 ± 4.93	20.22 ± 4.10	Group × Time	0.347
Sit-and-reach (cm)	Taekwondo	11.82 ± 7.64	12.91 ± 6.73	Group	0.545
				Time	0.658
	Control	9.05 ± 7.21	9.13 ± 6.77	Group × Time	0.066

Values are means ± standard deviations. *, **, *** indicate values that are significantly different from baseline: * *p* < 0.05, ** *p* < 0.01, *** *p* < 0.001.

**Table 4:** The changes in physical characteristics between the groups at baseline and after 12 weeks.

Variable	Group	Baseline	12 Weeks	Source	*p*-Value
Body weight (kg)	Taekwondo	55.95 ± 4.94	54.65 ± 4.38	Group	0.496
				Time	0.068
	Control	54.12 ± 3.26	53.83 ± 2.77	Group × Time	0.212
Body mass index (kg/m^2^)	Taekwondo	24.72 ± 2.10	24.14 ± 1.79	Group	0.073
				Time	0.108
	Control	24.20 ± 1.98	24.08 ± 1.83	Group × Time	0.220
Percent fat (%)	Taekwondo	42.41 ± 3.78	40.07 ± 3.69	Group	0.403
				Time	0.012 *
	Control	42.17 ± 5.26	43.46 ± 5.64	Group × Time	0.012 *
ASMI (kg/m^2^)	Taekwondo	5.34 ± 0.30	5.66 ± 0.40	Group	0.540
				Time	0.863
	Control	5.55 ± 0.14	5.26 ± 0.46	Group × Time	0.003 **
Systolic blood pressure (mmHg)	Taekwondo	148.82 ± 9.93	136.27 ± 8.04	Group	0.124
				Time	0.092
	Control	148.20 ± 14.03	151.01 ± 12.40	Group × Time	0.007 **
Diastolic blood pressure (mmHg)	Taekwondo	86.91 ± 8.22	76.27 ± 9.41	Group	0.607
				Time	0.000 ***
	Control	83.30 ± 8.72	83.54 ± 7.59	Group × Time	0.001 **
GDS-K (score)	Taekwondo	15.64 ± 1.75	13.18 ± 2.14	Group	0.003 **
				Time	0.689
	Control	16.00 ± 1.76	18.80 ± 2.10	Group × Time	0.000 ***
Positive items (score)	Taekwondo	8.73 ± 2.15	7.09 ± 2.59	Group	0.262
				Time	0.814
	Control	8.20 ± 1.75	10.00 ± 1.94	Group × Time	0.020 *
Negative (score)	Taekwondo	6.91 ± 2.02	6.09 ± 2.47	Group	0.116
				Time	0.945
	Control	7.80 ± 2.62	8.80 ± 2.15	Group × Time	0.184

Values are means ± standard deviations. *, **, *** indicate values that are significantly different from baseline: * *p* < 0.05, ** *p* < 0.01, *** *p* < 0.001. ASMI: appendicular skeletal muscle mass index; GDS-K: geriatric depression scale-Korea.

**Table 5:** The changes of dementia risk factors between the groups at baseline and after 12 weeks.

Variable	Group	Baseline	12 Weeks	Source	*p*-Value
TC (mg/dL)	Taekwondo	198.09 ± 26.44	165.00 ± 23.71	Group	0.070
				Time	0.173
	Control	199.20 ± 37.29	216.44 ± 32.59	Group × Time	0.000 ***
TG (mg/dL)	Taekwondo	156.09 ± 25.01	108.91 ± 25.95	Group	0.033
				Time	0.003 **
	Control	155.70 ± 19.52	156.57 ± 24.26	Group × Time	0.001 **
LDL-C (mg/dL)	Taekwondo	127.09 ± 23.52	101.91 ± 15.66	Group	0.099
				Time	0.067
	Control	127.77 ± 25.44	134.10 ± 19.33	Group × Time	0.001 **
HDL-C (mg/dL)	Taekwondo	53.64 ± 9.48	60.91 ± 8.83	Group	0.467
				Time	0.947
	Control	58.50 ± 16.83	48.79 ± 12.12	Group × Time	0.001 **
FFA (μEq/L)	Taekwondo	644.91 ± 313.95	535.18 ± 257.62	Group	0.016 *
				Time	0.929
	Control	724.50 ± 295.14	852.82 ± 242.15	Group × Time	0.012 *
Adiponectin (μg/mL)	Taekwondo	7.85 ± 2.18	9.99 ± 2.21	Group	0.026 *
				Time	0.956
	Control	8.29 ± 2.24	6.10 ± 6.36	Group × Time	0.002 **
Arteriosclerosis index	Taekwondo	2.79 ± 0.75	1.75 ± 0.46	Group	0.033
				Time	0.862
	Control	2.66 ± 1.16	3.71 ± 1.57	Group × Time	0.001 **
K-DSQ (score)	Taekwondo	5.73 ± 2.87	3.00 ± 1.67	Group	0.018 *
				Time	0.780
	Control	5.90 ± 2.88	8.50 ± 3.66	Group × Time	0.002 **
Irisin (μg/mL)	Taekwondo	8.14 ± 1.17	7.55 ± 1.47	Group	0.898
				Time	0.053
	Control	9.97 ± 3.82	7.20 ± 1.68	Group × Time	0.238
β-amyloid (pg/mL)	Taekwondo	3.69 ± 2.08	2.63 ± 0.86	Group	0.258
				Time	0.938
	Control	3.22 ± 0.85	4.36 ± 8.29	Group × Time	0.002 **
BDNF (pg/mL)	Taekwondo	24,134.67 ± 3889.04	29,933.33 ± 2626.50	Group	0.039
				Time	0.448
	Control	24,769.75 ± 7945.22	20,705.83 ± 3126.01	Group × Time	0.012 *

Values are means ± standard deviations. TC: Total cholesterol, TG: Triglyceride, LDL-C: Low density lipoprotein cholesterol, HDL-C: High density lipoprotein cholesterol, FFA: Free fatty acid, K-DSQ: Korean dementia screening questionnaire, BDNF: Brain-derived neurotrophic factor. *,** Significantly different from baseline: * *p* < 0.05, ** *p* < 0.01, *** *p* < 0.001.

## Data Availability

Qualified researchers can obtain the data from the corresponding author (ehk1959@dau.ac.kr and sgpark@dau.ac.kr). The data are not publicly available due to privacy concerns imposed by the IRB ethical principles.
